# Relative Abundance and Diversity of Bacterial Methanotrophs at the Oxic–Anoxic Interface of the Congo Deep-Sea Fan

**DOI:** 10.3389/fmicb.2017.00715

**Published:** 2017-04-25

**Authors:** Sandrine Bessette, Yann Moalic, Sébastien Gautey, Françoise Lesongeur, Anne Godfroy, Laurent Toffin

**Affiliations:** ^1^Institut Carnot Ifremer EDROME, Centre de Bretagne, REM/EEP, Laboratoire de Microbiologie des Environnements Extrêmes, UMR 6197Plouzané, France; ^2^Laboratoire de Microbiologie des Environnements Extrêmes, Institut Universitaire Européen de la Mer, UMR 6197, Université de Bretagne OccidentalePlouzané, France; ^3^CNRS, Laboratoire de Microbiologie des Environnements Extrêmes, Technopôle Brest Iroise, UMR 6197Plouzané, France

**Keywords:** aerobic methane-oxidizing bacteria, *pmoA*, organic-rich sediment, methane seeps, Congo deep-sea fan

## Abstract

Sitting at ∼5,000 m water depth on the Congo-Angola margin and ∼760 km offshore of the West African coast, the recent lobe complex of the Congo deep-sea fan receives large amounts of fluvial sediments (3–5% organic carbon). This organic-rich sedimentation area harbors habitats with chemosynthetic communities similar to those of cold seeps. In this study, we investigated relative abundance, diversity and distribution of aerobic methane-oxidizing bacteria (MOB) communities at the oxic–anoxic interface of sedimentary habitats by using fluorescence *in situ* hybridization and comparative sequence analysis of particulate mono-oxygenase (*pmoA*) genes. Our findings revealed that sedimentary habitats of the recent lobe complex hosted type I and type II MOB cells and comparisons of *pmoA* community compositions showed variations among the different organic-rich habitats. Furthermore, the *pmoA* lineages were taxonomically more diverse compared to methane seep environments and were related to those found at cold seeps. Surprisingly, MOB phylogenetic lineages typical of terrestrial environments were observed at such water depth. In contrast, MOB cells or *pmoA* sequences were not detected at the previous lobe complex that is disconnected from the Congo River inputs.

## Introduction

Deep-sea fans, also known as deep-sea turbidite systems, trap enormous amounts of terrestrial organic matter (OM) inputs delivered from continental shelves to deep ocean basins by rivers. Therefore, deep-sea fans are among the largest sedimentary systems on earth and contain large reservoirs of organic carbon buried in marine sediments (Shanmugam and Moiola, 1988). The Quaternary Congo deep-sea fan located on the Congo-Angola passive margin, is one of the largest mud-rich fans (300,000 km^2^) in the world and constitutes the major carbon burial region in the southeastern Atlantic Ocean (Rabouille et al., 2009). Due to its direct connection to the Congo River, the second largest river in the world in terms of fluvial discharge, continuous massive sedimentary inputs are transferred *via* turbidity currents following the path of the canyon deeply incised in the shelf (Khripounoff et al., 2003), regardless of sea level fluctuations (Droz et al., 2003). These sedimentary inputs flow 760 km off the Congo-Angola margin along the present-day active channel-levee system, which ends with lobe-shaped sedimentary deposits called the recent lobe complex (Savoye et al., 2009). The accumulated sediments (1.25 × 10^6^ t of organic carbon yr^-1^) of the recent lobe complex contain high organic carbon content (up to 5 wt% TOC; Baudin et al., 2017b) of terrestrial origin (70–80%; Stetten et al., 2015; Baudin et al., 2017b, which were deposited at extremely high sedimentation rates (>2–20 mm yr^-1^) (Stetten et al., 2015; Rabouille et al., 2016). As a consequence, substantial localized degradation of recent OM forms diffuse seepages enriched in methane (CH_4_) with hydrogen sulfide rising upward (Khripounoff et al., 2015) and a steep oxygen (O_2_) gradient at the sediment-water interface (Rabouille et al., 2009). These CH_4_-enriched sedimentary areas support remarkable chemosynthetic fauna on the seafloor that resemble the chemosynthetic communities associated with cold seeps (Rabouille et al., 2016).

The major biological sink of CH_4_ produced in sulfate-depleted anoxic sediments is anaerobic CH_4_ oxidation (Boetius and Wenzhofer, 2013). However, an unknown fraction of the CH_4_ rising upward through oxygenated sediments bypasses this benthic CH_4_ filter and is consumed by aerobic methane-oxidizing bacteria (MOB). MOB utilizes CH_4_ as their sole carbon and energy source at the sediment-water interface when oxygen from bottom waters is available as electron acceptor (Boetius and Wenzhofer, 2013). In disturbed seeps, such as the center of active Haakon Mosby Mud Volcano (HMMV) where anaerobic CH_4_ oxidation is repressed, or at Hikurangi Margin where the bioirrigation of the sediment-dwelling organisms enhances O_2_ advection in surface sediments (e.g., Hikurangi Margin), aerobic CH_4_ oxidation has been suggested to act as an efficient benthic filter regulating CH_4_ efflux at the surface sediments (Niemann et al., 2006; Thurber et al., 2013).

The first step of aerobic CH_4_ oxidation is performed by the particulate methane mono-oxygenase enzyme, encoded by the pmoCAB operon. All MOB described so far possess the canonical *pmoA* gene, with the exception of *Methylocella* spp. and *Methyloferula stellata* (Chen et al., 2010; Dedysh et al., 2015). The *pmoA* gene has been shown to be a relevant group-specific biomarker as its phylogeny is congruent to tree topology based on the 16S rRNA gene and it has been widely used in molecular studies of methanotrophs (McDonald et al., 2008).

The presence of MOB in marine methane-rich sediments, such as active mud volcano (Niemann et al., 2006), gas hydrates (Yan et al., 2006), carbonate mounds (Marlow et al., 2014) and in bottom waters of methane vent and seeps (Tavormina et al., 2008, 2010) has been demonstrated, as well as their abundance and activity (Lösekann et al., 2007; Steinle et al., 2015). These studies have revealed extensive undocumented and diverse phylogenetic lineages of MOB belonging mainly to *Gammaproteobacteria* (type I), which are present in these ecosystems, although *Alphaproteobacteria* (type II) have also occasionally been reported in shallow estuary sediments (McDonald et al., 2005).

The Congo deep-sea fan represents a new marine CH_4_-rich environment, which originates from the recycling of rich terrigenous organic carbon deposits in turbidite sediments and harbors peculiar habitats as well as biogeochemical processes similar to active cold seeps systems. The aims of the present study were (1) to identify and to quantify potential MOB at the oxic–anoxic interface of chemosynthetic habitats in five sites in Congo lobe complexes, (2) to assess whether the MOB communities vary between habitats, over the distal-proximal transect and across various ages of lobe complexes, (3) to compare phylogenetic diversity of MOB in the Congo deep-sea turbidite with a characteristic cold seep, and (4) to identify potential chemical variables shaping the MOB diversity and distribution.

## Materials and Methods

### Study Sites and Samples

**FIGURE 1:**
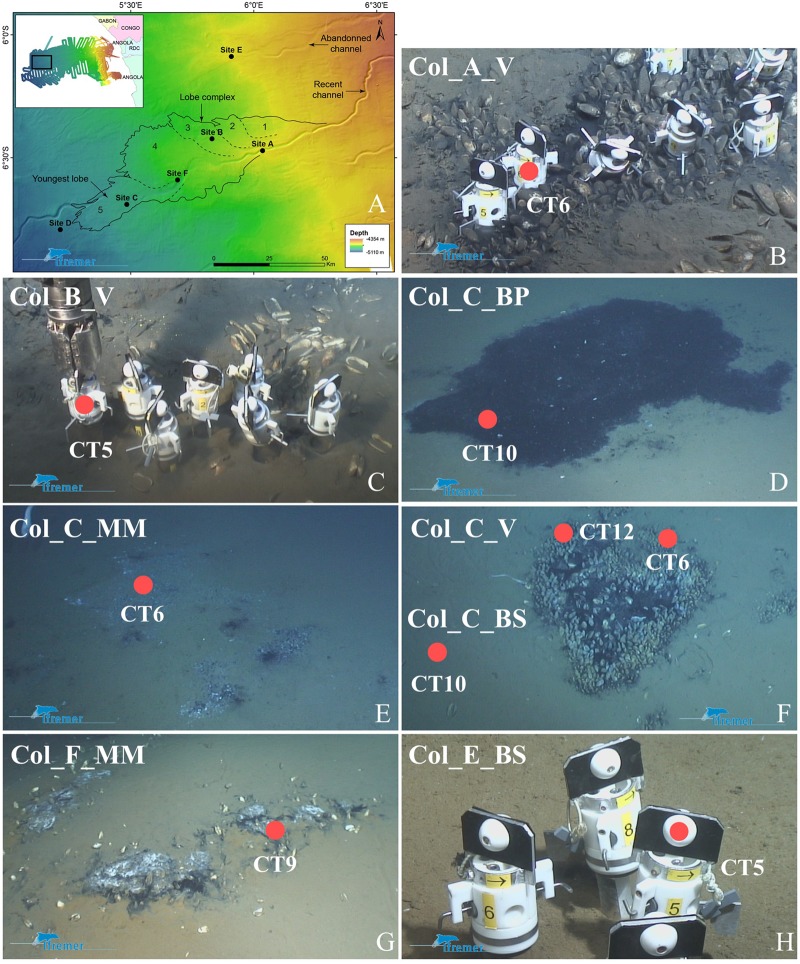
**Sampling sites and observed organic-rich habitats investigated in this study; adapted from (Rabouille et al., 2016). (A)** Bathymetric map of the recent lobe complex of the Congo turbidite system with the location of the five sites studied during the Congolobe cruise. The recent lobe complex of the Congo deep sea fan covers an estimated area of 3,000 km^2^ and is composed of a series of lobes (labeled 1–5 from the oldest to the youngest) having a grape-like prograding downstream organization. **(B–H)** Sampling sites into chemosynthetic habitats observed using ROV Victor 6000 and core-tube sampler. Refer to **Table 1** for label significance. **(B)** Reduced sediment at site A with vesicomyids clusters (Col_A_V). **(C)** Sparse vesicomyids at site B (Col_B_V). Ecosystems at site C : **(D)** Black patch of reduced sediment (Col_C_BP), **(E)** Microbial mat (Col_C_MM), **(F)** Vesicomyids clusters and adjacent brown sediment (Col_C_V). **(G)** Microbial mat at site F (Col_F_MM). **(H)** Brown sediment at site E (Col_E_BS).

The Congolobe cruise (Rabouille, 2011) investigated the recent lobe complex at the distal of the present-day active channel-levee system (**Figure 1**). The recent lobe complex is thus an active system and is made of five partly stacked lobes that have a grape-like prograding downstream organization. Therefore, each lobe is characterized by a chronosequence of decreasing age (4 ka to present) in the upstream to downstream orientation and lobes were labeled 1–5 along this sequence (**Figure 1**).

The sites studied were chosen according to (i) a distal-proximal transect from the active channel on the recent lobe complex (sites A, B, F, C) and (ii) lobe complexes ages: the recent lobe complex (sites A, B, F, C) and the previous lobe complex (site E) aged between 6 and 4 ka BP (Picot et al., 2016) (**Figure 1**). Sediments were recovered within various types of dense chemosynthetic habitats at different sites between 4644 and 4947 m water-depth using the ROV Victor 6000 (Ifremer) core-tube sampler aboard the R/V Pourquoi Pas? (**Figures 1B–H**).

The description of the studied sites is summarized below (**Table 1**) and further information is provided in Rabouille et al. (2016):

**Table 1:** Origin of the sites and samples investigated in this study.

Sites	Habitats – Markers	Dive	Core tubes	Latitude longitude	Water depth (m)	Sample name
Site A	Reduced sediment with vesicomyids clusters (V) (Col2 marker)	PL484-4	CT6	S 06 28.2798 E 006 02.137	4769	Col_A_V
Site F	Microbial mat (MM) with sparse vesicomyids (Col3 marker)	PL486-6	CT9	S 06 35.4307 E 005 41.412	4872	Col_F_MM
Site C	Microbial mat (MM) (Col4 marker)	PL490-10	CT6	S 06 41.3977 E 005 28.773	4947	Col_C_MM
	Black patch sediment (BP) (Col4 marker)	PL490-10	CT10	S 06 40.9606 E 005 28.921	4845	Col_C_BP
	Vesicomyids clusters (V) (Col4 marker)	PL491-11	CT6	S 06 42.0844 E 005 29.293	4946	Col_C_V_CT6
	Vesicomyids clusters (V) (Col4 marker)	PL491-11	CT12	S 06 42.0876 E 005 29.291	4846	Col_C_V_CT12
	Adjacent brown sediment (BS), surrounding the vesicomyids cluster (Col4 marker)	PL491-11	CT10	S 06 42.0779 E 005 29.292	4846	Col_C_BS
Site B	Sparse vesicomyids (V) (Col11 marker)	PL492-12	CT5	S 06 25.2301 E 005 49.708	4718	Col_B_V
Site E	Brown sediment (BS) (ColE494-dune01 marker)	PL494-14	CT5	S 06 05.5487 E 005 54.489	4644	Col_E_ BS
Haakon Mosby mud volcano	Center of the mud volcano	–	–	N 720.2784 E 1443.5639	1273	VKGMTB8

*During the Congolobe cruise (R/V Pourquoi Pas? December 2011–January 2012), sediments were recovered within various types of dense chemosynthetic habitats at different sites between 4644 and 4947 m water-depth using the ROV Victor 6000 (Ifremer) core-tube sampler. During the Viking cruise (R/V Pourquoi Pas? June 2006) sediment was sampled at the center of the Haakon Mosby Mud Volcano (HMMV) by a multicorer MUC (VKGMTB8) at about 1250 m water depth. PL, Dive number; CT, core tubes number; Col, **Co**ngo **l**obe, physical markers were deployed at all the investigated habitats of the Congo lobes. V, vesicomyids bivalves; BP, black patch; BS, brown sediment; MM, microbial mat.*

• Site A is located at the entrance of the recent lobe complex, which is also the entrance of the oldest lobe (#1) and was colonized by vesicomyid bivalve patches edging black reduced sediments (Col_A_V).
  Site B is located 22 km northwestward from site A in lobe (#3). It is abandoned from the active channel but still receives material from the turbiditic current overflow during important episodic turbiditic events (Bonnel, 2005). Black reduced sediments patches dominated by sparse vesicomyid bivalve’s shells and disarticulated valves were observed (Col_B_V).
• Site F is in the active-channel located 40 km downstream of site A, at the entrance of the youngest lobe (#5). It was characterized by thin grayish-whitish mats surrounding black reduced sediments (Col_F_MM).
• Site C is located 25 km from site F in the distal part of the youngest lobe (#5). It was characterized by the occurrence of diverse habitats: reduced sediments, defined by black patches with a few empty shells of vesicomyid bivalves (Col_C_BP), white microbial mat area surrounding black reduced sediment (Col_C_MM), dense vesicomyid bivalve’s colonies at the periphery of black reduced sediment patches (Col_C_V_CT6 and Col_C_V_CT12) characterized by dense populations of ampharetids and adjacent brown and bare sediments (Col_C_BS).
• Site E is located approximately 45 km to the north of site A and explored the feeding channel of the previous lobe complex, which is disconnected from the active channel and is interpreted as having been abandoned for several 1000 years. No seafloor biological manifestation was observed. One core-tube (Col_E_BS) sampled at this site, provides a reference for the onset to the hemipelagic sedimentation after the interruption of turbiditic deposition.

At each location, separate sediment core-tubes were collected for microbiological and geochemical analyses.

After retrieval, the cores were transferred to the cold room at 4°C and were sliced at intervals in 2-cm-thick layers. In this study, the first centimeters (0–2 cm) were analyzed and corresponded to the oxic–anoxic interface at shallow sediments. Sediment subsamples for nucleic acid extractions were frozen at -80°C. One cm^3^ of sediment subsample was fixed in PBS (phosphate-buffered saline)/formaldehyde (3% final), washed twice with PBS, and finally stored in PBS/Ethanol (1:1, vol:vol) at -20°C for fluorescence *in situ* hybridization (FISH) (Pernthaler et al., 2001).

In order to partially answer one of the questions, MOB communities in deep-sea turbidite system were compared with an additional sediment sample collected in 2006 from the active center of the HMMV, where freshly expelled fluid and mud support dense populations of MOB communities (Niemann et al., 2006; Lösekann et al., 2007). Briefly, this sediment was sampled by a multicorer MUC (VKGMTB8) at about 1250 m water depth on the slope of the Barents Sea continental margin (VICKING Cruise, R/V Pourquoi Pas? June 2006) (Nouze, 2006). The core was stored at 4°C and sliced at intervals in 2-cm-thick layers. In this study, similarly the first centimeters (0–2 cm), corresponding to the oxic–anoxic interface was analyzed and sediment subsamples were frozen at -80°C for nucleic acid extraction.

### Preparation of Cell Staining and Fluorescence *In Situ* Hybridization

Cell staining and FISH reactions were performed for four selected samples at site C in the recent lobe complex (Col_C_MM, Col_C_BP, Col_C_V_CT6) and one reference sample at site E from the previous lobe complex (Col_E_BS).

Ten μL of a 100-fold dilution of fixed sediment subsamples, were gently mixed with a Vortex-Genie^®^ 2 Mixers (Scientific Industries) to ensure that cells were in suspension and decanted and kept on ice for 10–15 min prior to transferring 40 μL of the supernatant into 8 mL of PBS. Samples were filtered through a 0.22 μm GTTP polycarbonate filter (Merck Millipore, Darmstadt, Germany) and air-dried.

*In situ* hybridization conditions were optimized to maximize efficiency and specificity of fluorescence signal. An optimal signal was obtained with 40% formamide. The specificity of the probes was further checked on a pure culture of *Methylomarinum vadi* strain IT-4^T^ (Hirayama et al., 2013). Dual hybridizations with the Alexa-labeled EUB338 probe (16S rRNA; positions 338–355) for members of the domain *Bacteria* (Amann et al., 1990) and either a mix of Cy3-labeled 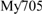 and 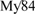 (16S rRNA; positions 84–101 and positions 705–722) probes or Cy3-labeled Mα450 (16S rRNA; positions 450–469) for the detection of Type I and Type II MOB cells, respectively (Eller et al., 2001) as well as Cy3-labeled MTMC-701 (16S rRNA; positions 701–718) for most members of the order *Methylococcales* (Boetius, 2000) were used (Supplementary Table 2).

Filters were hybridized in a reaction mix containing 0.5 μM of each probe in a formamide hybridization buffer [0.9 M NaCl, 0.02 M Tris-HCl, 0.01% sodium dodecyl sulfate (SDS), 40% formamide] for 3 h at 46°C. Filters were then washed for 20 min at 48°C in a washing buffer [NaCl, 0.02 M Tris-HCl, 0.004 M EDTA, 0.01% SDS], rinsed briefly with sterile MilliQ water and air-dried (Pernthaler et al., 2001). Cells were counterstained with 10 μL of 4′, 6′-diamidino-2-phenylindole (DAPI; 1 μg/mL in MilliQ; 10 μL) for 10 min, briefly rinsed with sterile MilliQ water and left to air-dry. Finally, filters were fixed on microscopic slides and embedded in Citifluor AF1 (Citifluor, Ltd, UK) and stored at -20°C.

### Procedure for Single Cells and Aggregated Cells

Single cells were counted after *in situ* hybridization by epifluorescence microscopy according to the method described by Goulder (1977). Triplicate filters for all samples were prepared for each dual hybridization conditions to minimize count variability and were subsequently counted in 100 independent microscopic fields. Blank samples were processed by treating 8 mL of 0.2 μm filtered PBS solution. Aggregate cells were counted as described by Lösekann et al. (2007). Observations, enumerations, and images were performed with a Zeiss Axio Imager 2 epifluorescence microscope (Carl Zeiss) equipped with the slider module ApoTome^®^ (Zeiss) at 100X magnification, the illumination system HXP-X and Colibri light technology (Zeiss) with appropriate filter sets for Cy3, Alexa Fluor 488, GFP, and DAPI fluorescence using an AxioCam MRm camera (Zeiss). Epifluorescence acquisitions were analyzed using the ZEN Pro 2012 software (Zeiss).

### DNA Extraction

Total nucleic acids were directly extracted from 3 to 5 g of wet sediments using the PowerMax^®^ Soil DNA Isolation Kit (MO BIO Laboratories, Inc., Carlsbad, CA, USA) according to the manufacturers’ instructions. DNA was purified and concentrated by adding 0.2 mL of NaCl 5 M (SIGMA, St. Louis, MO, USA) and 10.4 mL of cold absolute ethanol (CARLOS EBRA, Val de Reuil, France). Samples were incubated 10 min at room temperature following by 5 min on ice, and then centrifuged for 30 min at 2,500 *g* at room temperature. The DNA pellets were washed with 5 mL of 70% ethanol and left to air-dry. The pellets were suspended in 200 μL of sterilized MilliQ water overnight at 4°C. Purified DNA extracts were stored at -20°C.

### PCR, Clone Libraries, and Sequencing

Bacterial 16S rRNA genes were amplified by polymerase chain reaction (PCR) using the primer set E8F (5′-AGAGTTTGATCATGGCTCAG-3′) and U907R (5′-CCGTCAATTCMTTTRAGTTT-3′) (Lane et al., 1985). *PmoA* genes were amplified using the primer set A189f (5′-GGNGACTGGGACTTCTGG-3′) and mb661r (5′-CCGGMGCAACGT CYTTACC-3′) (Holmes et al., 1995; Costello and Lidstrom, 1999). These primers provide the widest coverage of the known *pmoA* diversity (McDonald et al., 2008) and are known to discriminate against sequences of the *amoA* genes (Dumont and Murrell, 2005); a *pmoA* homolog encoding the α-subunit of ammonia mono-oxygenase.

Polymerase chain reactions were performed in independent triplicates and PCR master mix consisted of 1X Green Go*Taq*^®^Flexi Buffer (Promega, Madison, WI, USA), 2 mM MgCl_2_ (Promega, Madison, WI, USA), 0.4 mM dNTP mix, 0.2 μM of each primer (Eurogentec, Liège, Belgique), 0.6 U of Go*Taq*^®^Flexi DNA polymerase (Promega, San Luis Obispo, CA, USA) and 1 μL of purified DNA template and sterile water to a final volume of 25 μL.

The PCR protocol consisted of an initial 95°C denaturing step for 5 min, followed by 30 cycles of 95°C for 45 s, 56°C for 1 min and 72°C for 45 s for *pmoA* genes or 94°C for 1 min, 57°C for 1 min 30 and 72°C for 2 min for bacterial 16S rRNA genes, and then a final 72°C elongation step for 6 min. Absence of contaminations was checked by negative controls. All PCR reactions were carried out using a GeneAmp^®^ PCR system 9700 (Applied Biosystems, Foster City, CA, USA) thermal cycler, and PCR products were visualized using gel electrophoresis.

Amplified 16S rRNA and *pmoA* gene fragments from pooled triplicate reactions were purified on TAE agarose gels (0.8%) using NucleoSpin^®^ Gel and PCR^®^ Clean-up kits (Macherey Nagel, Düren, Germany) according to the manufacturer’s instructions. The purified amplicons were ligated into pCR2.1-TOPO vector (Invitrogen, Carlsbad, CA, USA) and transformed into *Escherichia coli* X-Gold^®^ chemically competent cells (Stratagene) according to the manufacturer’s recommendations. Cloned *pmoA* fragments were sequenced using M13 universal primer with BigDye terminator chemistry and determined on an ABI3730xl Genetic Analyser (Applied Biosystems) (GATC Biotech, Konstanz, Germany).

The quality of retrieved 16S rRNA and *pmoA* sequences chromatograms and base calls were manually verified using Geneious© R6 version 6.1.7 (Kearse et al., 2012) and the vector and priming sites were excluded. Sequences were also screened for the closest relatives using blastn against the NCBI NR database. A customized *pmoA* database was created from sequences available in the NCBI database. Sequences were imported into a seed alignment of the ARB software package (Ludwig et al., 2004) and *pmoA* sequences were translated into amino acid sequences.

### Assignment of OTUs, Diversity Metrics, and Statistics

Lower triangular distance matrices were calculated based on a SAI filter including 150 deduced amino acid positions using the neighbor-joining method (Saitou and Nei, 1987) and corrected with the PAM model (Wilbur, 1985) in ARB. The *pmoA* matrices were exported to Mothur (version 1.36.0) (Schloss et al., 2009) and sequences were clustered into operational taxonomic units (OTUs) using the average linkage algorithm with a cutoff distance of 7%. The PmoA distance of 7% was demonstrated to correspond to the 3% 16S rRNA distance, thus representing an indication for a representative methanotroph species (Lüke and Frenzel, 2011). Representative sequences of calculated OTUs (OTU-rep) were used for further in-depth phylogenetic analyses. OTU-rep is a sequence that had the minimum distance to the other closest sequences of the same OTU as calculated in Mothur (Schloss et al., 2009). Richness (Chao), Shannon -alpha diversity indices, as well as rarefaction curves, were calculated using Mothur (Schloss et al., 2009).

Analysis of variance (ANOVA) was performed with PAST software (Hammer et al., 2001) to test for significant contributions of MOB abundances between habitats.

To investigate patterns in the MOB community structure of the Congo lobe sediments and the link with selected chemical parameters (Supplementary Data and Table 1), we performed a canonical correspondence analysis (CCA) to determine parameters that could best explain the MOB community structure. The significance of the models was tested by analysis of variance with 1000 permutations. Co-inertia analysis (CoIA) was performed as well to identify trends or co-relationships in multiple datasets. A Monte Carlo permutation test (999 times) was performed to assess the significance of the co-structure of the data tables. Chemical data (CH_4_ concentration and efflux, diffusive oxygen uptake, oxygen penetration depth) were standardized to zero mean and unit variance before analysis. CoIA and CCA were done with the Ade4 and vegan packages (Thioulouse et al., 1997; Oksanen et al., 2013).

### Phylogenetic Analysis

The 16S rRNA nucleic acid and PmoA amino acid gene sequences were aligned with Clustal W (Thompson et al., 1994) implemented in ARB and the alignments were refined manually. Phylogenetic trees were generated from an observed distance matrix using the neighbor-joining method and the partial adjustment model (PAM) correction (Wilbur, 1985) using ARB. The robustness of inferred topologies was calculated by bootstrap analyses with 100 resamplings of trees. Phylogenetic analyses were used to assign taxonomic for all *pmoA* sequences and to plot phylogenetic trees of OTU-rep.

### Bipartite Network

A bipartite network was constructed to investigate co-occurrence patterns of MOB between samples. The two categories of node coexisting in this network correspond to the OTUs (circles) and the samples (squares). The network is based on Jaccard’s distance at the OTU level, which means that links denote the OTU memberships presents within samples. We visualized and computed the eigenvector centrality (ranging from 0 to 1) of the network with Gephi (Bastian et al., 2009). This measure assesses how influential a node is in the network topology through the number of connections weighted by the degree of its neighbors (Bonacich, 2007).

### Nucleotide Sequence Accession Numbers

The nucleotide sequence data reported in this paper were submitted to GenBank/EMBL/DDBJ sequence databases under accession numbers KY350874–KY351540.

## Results

### Relative Abundances of *Bacteria* and MOB in Sedimentary Habitats of the Congo Lobe Complexes

Relative bacterial abundances detected by FISH were higher in chemosynthetic habitats (ranged from 1.3 to 4.2 × 10^7^ cell/ml) of the recent lobe complex than in adjacent sediment (Col_C_BS) of the same lobe complex (0.1 ± 0.0 × 10^7^ cell/ml) or than in sediment from the previous lobe complex (Col_E_BS; 0.1 ± 0.0 × 10^7^ cell/ml) (**Table 2**). Black patch sediment (Col_C_BP) contained the highest relative number of bacterial cells, which mainly occurred as aggregates (**Table 2**).

**Table 2:** Fluorescence *in situ* hybridization (FISH) quantification of bacteria and methane-oxidizing bacteria (MOB) at the oxic–anoxic interface of sedimentary habitats at site C from the recent lobe complex (active system) and at site E from the previous lobe complex (abandoned system) of the Congo deep-sea fan.

Samples	Total bacterial cell abundance (10^7^/mL)	Type I MOB cell abundance (10^6^/mL)	*Methylococcales* cell abundance (10^6^/mL)	Type II MOB cell abundance (10^6^/mL)	Total MOB cell abundance (10^6^/mL)
Col_C_MM	1.31 ± 0.43	1.10 ± 0.28	1.01 ± 0.16	0.69 ± 0.30	1.79 ± 0.41
Col_C_BP	4.27 ± 1.01	2.10 ± 0.15	1.96 ± 0.06	0.75 ± 0.17	2.85 ± 0.22
Col_C_V_CT6	1.93 ± 0.18	0.61 ± 0.23	0.74 ± 0.05	0.27 ± 0.19	0.88 ± 0.29
Col_C_BS	0.15 ± 0.03	0.32 ± 0.14	0.36 ± 0.16	0.17 ± 0.03	0.49 ± 0.14
Col_E_BS	0.10 ± 0.00	0.00 ± 0.00	0.00 ± 0.00	0.00 ± 0.00	0.00 ± 0.00


*Col, Congo lobe; V, vesicomyids bivalves; B, black patch sediment; BS, brown sediment; MM, microbial mat.*

In sediments from the previous lobe complex (Col_E_BS), neither MOB cells were observed (**Table 2**) nor CH_4_ efflux detected (Supplementary Table 1). In contrast, MOB cells were detected in all sediments from habitats at site C from the recent lobe complex (**Table 2**). The highest relative number of MOB was observed in black patch sediment (Col_C_BP; 2.8 ± 0.2 × 10^6^ cell/ml) while the lowest MOB abundance was observed in adjacent sample (Col_C_BS; 0.49 ± 0.1 × 10^6^ cell/ml). The ratio between relative MOB and bacteria abundances ranged from 4 to 33% depending on the habitats. Type I abundances were higher in black patch sediment and microbial mat habitat while type II abundances were in the same order of magnitude in all samples (**Table 2**). The contribution of types I and II suggested a trend (one-way ANOVA; *F* = 3.9, *p* = 0.07) of higher abundances of type I cells (up to 2 × 10^6^ cells mL^-1^, 61–74% of MOB mainly represented by members of the order *Methylococcales*) compared with type II (up to 0.7 × 10^6^ cells mL^-1^, 26–38% of MOB).

**FIGURE 2:**
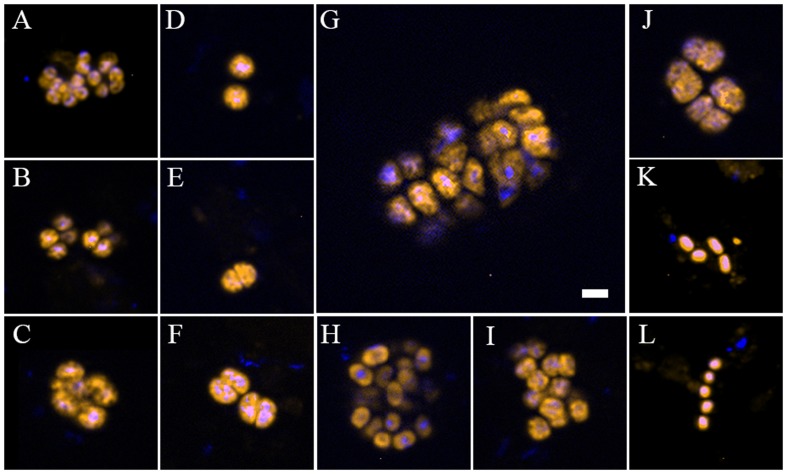
**Individual cells and cell aggregates of methane-oxidizing bacteria (MOB) at the oxic–anoxic interface of organic-rich sedimentary habitats at the recent lobe complex (site C) of the Congo deep-sea fan, visualized with specific fluorescent-labeled oligonucleotides probes. (A–C)** Clusters of type I MOB detected with the specific Cy3-labeled probe Mγ84/705 in parallel with DAPI staining. **(D–J)** Shape, spatial organization, and size of *Methylococcales* (Cy3-labeled probe MTMC-701) in parallel with DAPI staining. **(K,L)** Type II MOB cells hybridized with Cy3-labeled probe Mα450 in parallel with DAPI staining. Image A was taken from a sample collected at microbial mat habitats (Col_C_MM); **(B,C,D–L)** from black patch habitat (Col_C_BP). The scale bar represents 2 μm length.

Type II-MOB cells occurred as single/double cells or multicellular colonies and were rod- or coccoid-shaped of ∼0.5–2 μm in size and free-living (**Figures 2K,L**). In contrast, type I-MOB cells occurred as double cells or paired- multicellular colonies and differed in morphology as large coccoid- or pear-shaped cells of ∼1–2 μm diameter (**Figures 2A–J**).

### Richness and Diversity of MOB in Congo Lobe Sedimentary Habitats

**FIGURE 3:**
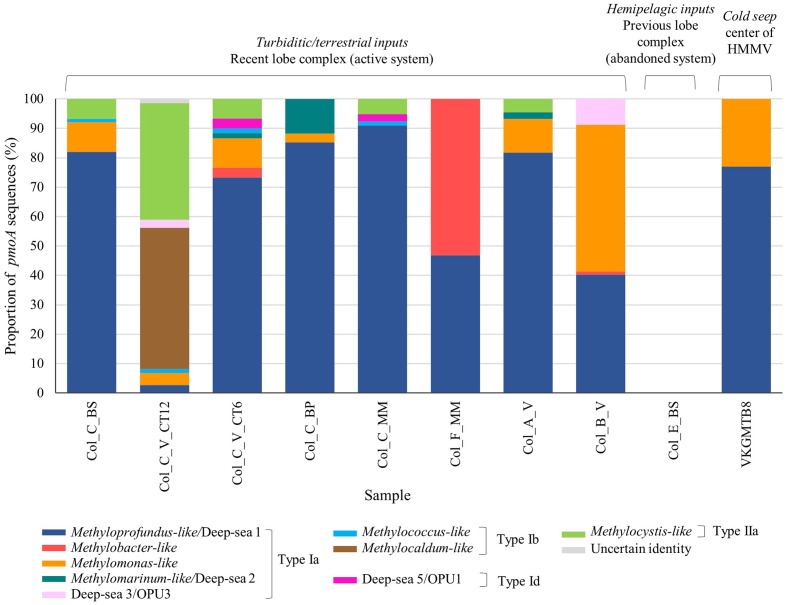
**Phylogenetic affiliations and proportion of the pmoA amino acid sequences retrieved at surface organic-rich and seep sediments of habitats in the Congo lobe deep-sea fan (Col) and Haakon Mosby volcano (VKG) respectively based on neighbor-joining tree constructed using the ARB package and inferred with a PAM correction and 100 bootstrap replicates (158 curated amino acid positions)**.

To get further insights into the diversity of MOB communities, we analyzed 16S rRNA and *pmoA* genes between sedimentary habitats of the recent lobe complexes (active system) and the previous lobe complex (abandoned system) (**Figure 1**). None of the presumed MOB representative sequences were detected in 16S rRNA gene clone libraries. However, genes encoding *pmoA* were detected by PCR in all DNA samples from the recent lobe complex, yielding a total of 667 accurate sequences that were classified into 28 OTUs (**Table 3**). In all attempts, no *pmoA* gene fragment could be amplified by PCR from DNA of the sediment from the previous lobe complex (Col_E_BS) (**Figure 3**), which is consistent with FISH observations (**Table 2**).

**Table 3:** Number of *pmoA* amino acid sequences, richness, diversity, and characteristics of operational taxonomic units (OTUs) of *pmoA* amino acid sequences at a cutoff value of 0.07.

Samples	*pmoA* amino acid sequences	*pmoA* OTU_0.07_	*pmoA* OTU_0.07_ singletons	Unique *pmoA* OTU_0.07_	Shared *pmoA* OTU_0.07_	Chao1	Shannon Index (H′)
Col_A_V	88	12	5	3	9	15 (13, 34)	2
Col_B_V	92	11	2	3	8	12 (11, 19)	2
Col_C_BP	34	5	1	0	5	5 (5, 0)	1
Col_C_MM	78	11	2	0	11	11 (11, 17)	2
Col_C_BS	89	11	4	1	10	14 (11, 34)	2
Col_C_V_CT12	73	10	3	4	6	12 (10, 22)	1
Col_C_V_CT6	60	10	2	0	10	10 (10, 15)	2
Col_F_MM	79	8	0	0	8	8 (8, 8)	2
Col_E_BS	0	0	0	0	0	0	0
VKGMTB8	74	4	0	0	4	4 (4, 4)	1


*Chao1, Chao true diversity estimator. Number in brackets indicated the lower and upper bound of 95% confidence interval.*

Comparisons using the rarefaction curves showed that our sequencing effort did not cover the full *pmoA* diversity, with exceptions of the microbial mat habitat (Col_F_MM) and the black patch sediment (Col_C_BP) from the youngest lobe (#5), as well as the cold seep sample, HMMV (VKGMTB8) (Supplementary Figure 1). The richness estimates (Chao1) and rarefaction curves indicated that the highest richness was found in the vesicomyid habitat at site A (Col_A_V) (**Table 3** and Supplementary Figure 1). In contrast, both analyses showed that the lowest richness was found in the black patch sediment of site C (Col_C_BP) and in the cold seep sediment, HMMV (VKGMTB8) (**Table 3** and Supplementary Figure 1). These two samples with one of the two sediment samples from vesicomyids habitat at site C (Col_C_V_CT12) exhibited the lowest Shannon diversity index (H′) (**Table 3**).

### Phylogenetic Diversity of MOB Communities in Congo Lobe Sedimentary Habitats

Phylogenetic analysis of the *pmoA* sequences revealed a great diversity of four subdivided phylotypes Ia, Ib, Id, and type IIa in the sedimentary habitats of the recent lobe complex (**Figure 3**). With the exception of one sediment sample of the vesicomyid habitat (Col_C_V_CT12), all sediments were composed of sequences affiliated to *Methyloprofundus-*like clade (type Ia), a marine strain of the deep-sea 1 clade (Tavormina et al., 2015). Other deep-sea clades (2, 3/OPU3, and 5/OPU1) were found in very low relative proportion (less than ∼10%) in some samples (**Figure 3**). In contrast, the sediment sample from the vesicomyid habitat (Col_C_V_CT12) was dominated by sequences affiliated to *Methylocaldum*-like (type Ib) and to *Methylocystis*-like (type IIa) clades. Interestingly, the microbial mat habitat at site F (Col_F_MM) and vesicomyid habitat at site B (Col_B_V) were respectively dominated by sequences closely related to *Methylobacter*-like and to *Methylomonas*-like clades respectively.

In comparison to sediments of the recent lobe complex from the Congo turbidite system, sediment of the typical cold seep HMMV exhibited lower *pmoA* diversity and was represented only by two clades of type Ia: *Methyloprofundus*-like and *Methylomonas*-like (**Figure 3**).

**FIGURE 4:**
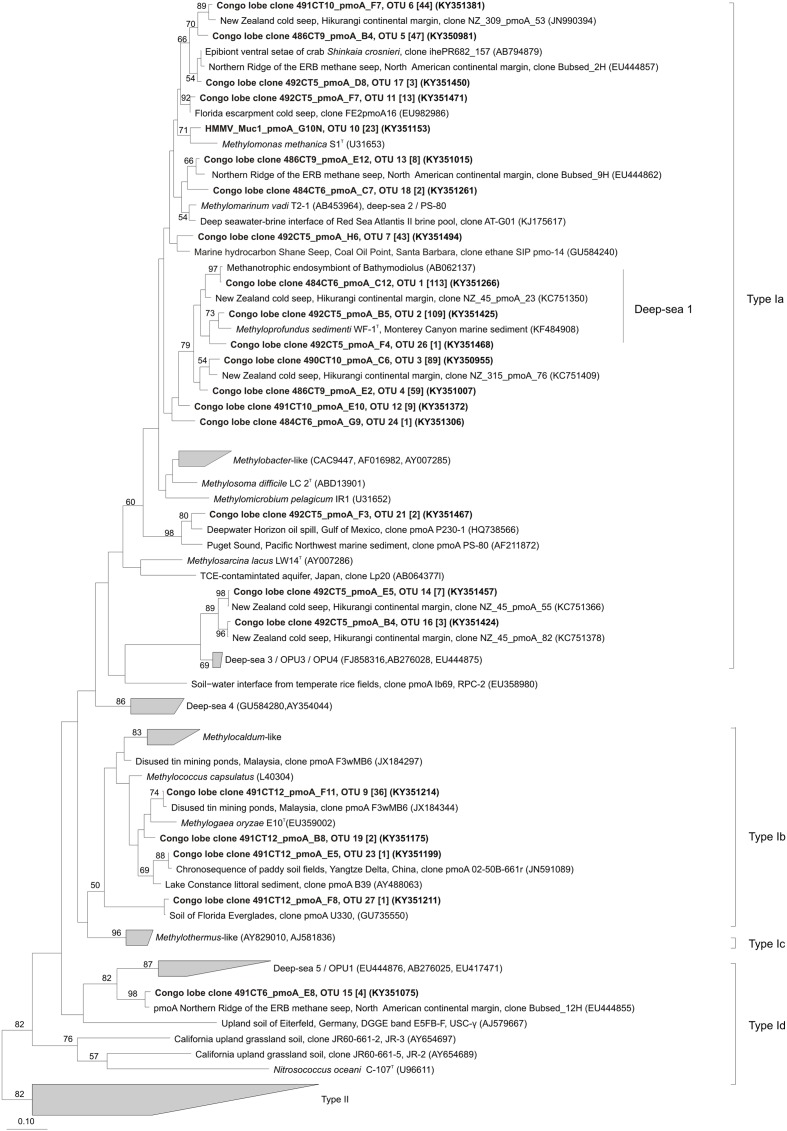
**Phylogenetic analysis of the inferred amino acid sequences encoded by the pmoA gene of type I methane-oxidizing bacteria.** The neighbor-joining tree was constructed using the ARB package and inferred with a PAM correction and 100 bootstrap replicates (158 curated amino acid positions). Bootstrap values higher than 50% are indicated. Scale bar represents 10% estimated substitutions per amino acid position. Representative sequences for each OTUs from this study are depicted in black bold and the corresponding number of sequences in OTUs are indicated in brackets.

**FIGURE 5:**
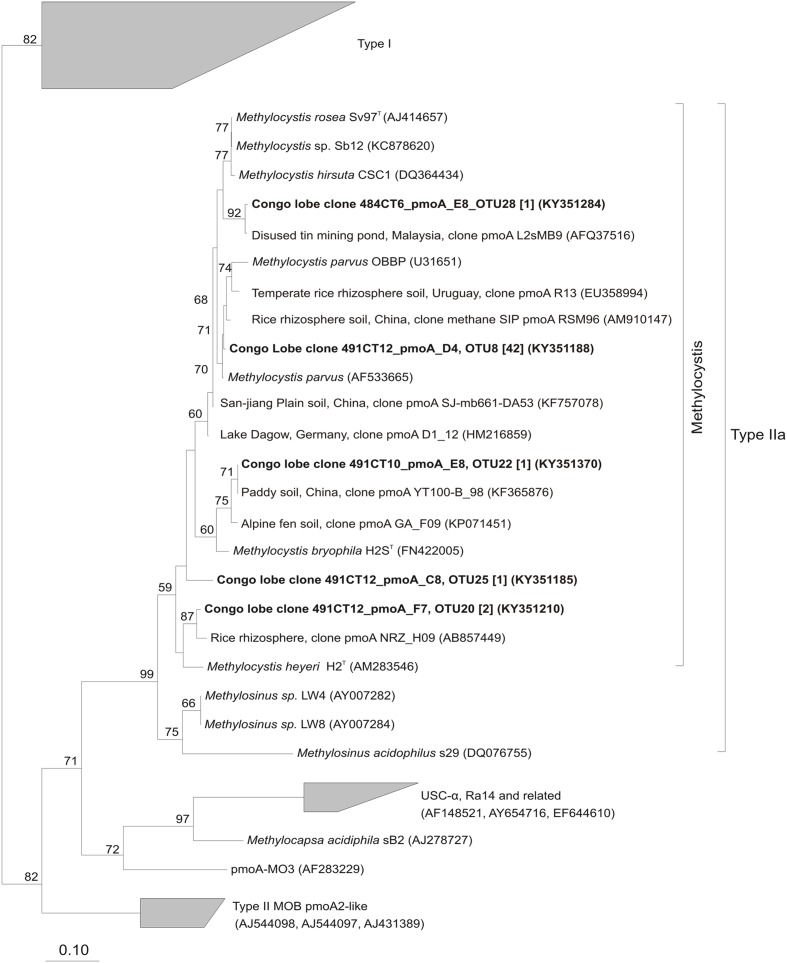
**Phylogenetic analysis of the inferred amino acid sequences encoded by the *pmoA* gene of type II methane-oxidizing bacteria.** The neighbor-joining tree was constructed using the ARB package and inferred with a PAM correction and 100 bootstrap replicates (158 curated amino acid positions). Bootstrap values higher than 50% are indicated. Scale bar represents 10% estimated substitutions per amino acid position. Representative sequences for each OTUs from this study are depicted in black bold and the corresponding number of sequences in OTUs are indicated in brackets.

Phylogenetic analysis indicated that OTUs were similar to *pmoA* sequences retrieved from both marine and terrestrial environments (**Figures 4**, **5**). The majority of the OTUs affiliated to type Ia fell into environmental clusters composed only of *pmoA* marine sequences isolated from methane seeps (Reed et al., 2009; Redmond et al., 2010; Kessler et al., 2011). In particular, most of the abundant OTUs were highly similar (90–100%) and closely affiliated to *pmoA* sequences recovered from a New Zealand cold seep at Hikurangi margin (Ruff et al., 2013) and to the cultivated genus *Methyloprofundus sedimenti* WF1^T^ (**Figure 4**). Within type Ib (**Figure 4**), sequences showed a close relationship to strain *Methylogaea oryzae* E10^T^ and grouped within a second environmental cluster of terrestrial sequences collected from soil and freshwater lake sediments (Ho et al., 2011a; Chauhan et al., 2012; Sow et al., 2014). Unique *pmoA* sequence affiliated to type Id (**Figure 4**) was related to a *pmoA* sequence retrieved from methane seeps from the North American margin and grouped within the deep-sea 5/OPU1 clade, supported with strong bootstrap values (Tavormina et al., 2008, 2010). Finally, within type IIa, five OTUs were closely related to the *Methylocystis-*like clade and *pmoA* sequences collected from rice paddies (Qiu et al., 2008; Bao et al., 2014) (**Figure 5**).

### Co-occurrence of MOB Communities and Factors Shaping Their Structure

**FIGURE 6:**
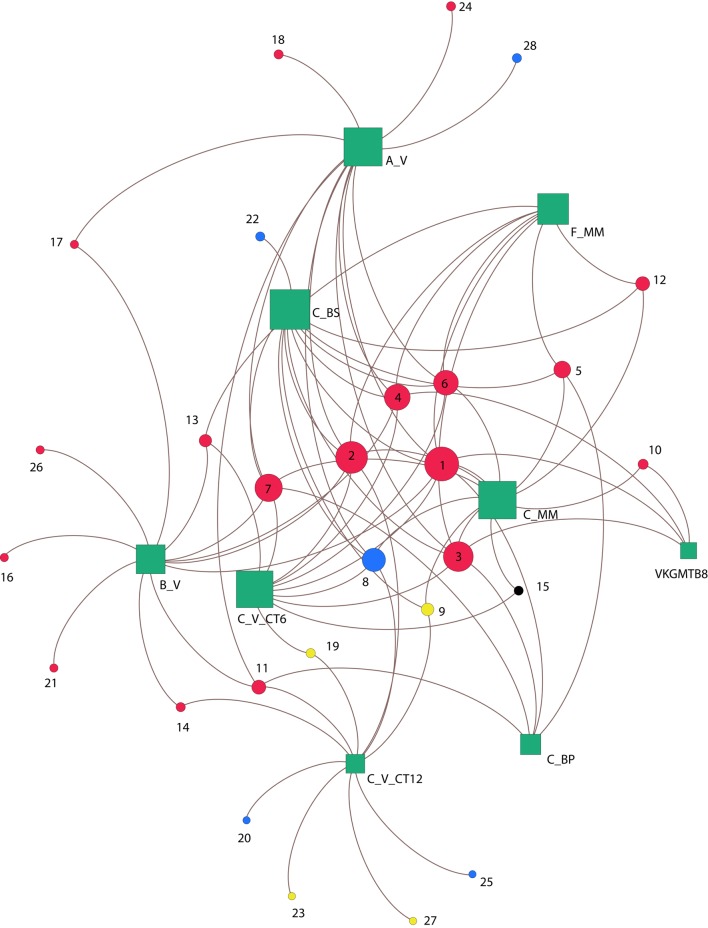
**Co-occurrence of *pmoA* OTUs between sites.** Green squares represent sedimentary habitats and circles correspond to OTUs memberships detected within habitats, based on the Betweenness-Centrality property. Node sizes are proportional to the number of connections. Colors circles indicate OTUs methane-oxidizing bacteria types (type Ia red; type Ib yellow; type Id black; and type IIa blue). Numbers indicates OTU names. Congo lobe habitats are labeled as follow: Col_C_MM (C_MM); Col_C_BP (C_BP); Col_C_V_CT6 (C_V_CT6); Col_C_V_CT12 (C_V_CT12); Col_C_BS (C_BS); Col_F_MM (F_MM); Col_A_V (A_V); Col_B_V (B_V). Haakon Mosby mud volcano is represented by sample VKGMTB8.

To study the associations between OTUs based on *pmoA* genes and their habitats, a network was constructed by computing the eigenvector centrality (**Figure 6**). Overall, none of the OTUs occurred at all sites, revealing heterogeneity in their distribution among the habitats. The center of the network is composed of a group of six influential OTUs: OTU 1, OTU 2, OTU 3, OTU 4, OTU 6, OTU 8 (having eigenvector centrality values higher than 0.5). All these taxa belong to type Ia (87.5%) except one of type IIa (OTU 8; 12.5%) (**Figure 6**), and were shared differentially between all samples as illustrated by the short paths. Among the major type Ia, the most abundant OTUs were OTU 1 (21% of *pmoA* sequences), OTU 2 (20% of *pmoA* sequences), OTU 3 (16% of *pmoA* sequences) and they were related to the deep-sea 1 clade (Lüke and Frenzel, 2011) (**Figures 4**, **6**).

The sediments from the cold seep HMMV (VKGMTB8) shared distantly (long paths) three of the most abundant type Ia OTUs (OTU 1, OTU 3, OTU 4) with samples of the recent lobe complex from the Congo turbidite system.

Likewise, with exception of one sediment sample of the vesicomyid habitat (Col_C_V_CT6), all the vesicomyid habitats showed the occurrence of unique OTUs (39%) (**Figure 6**). These unique OTUs belonged to the type Ia (18%), type IIa (14%) and few were associated to type Ib (7%) as well. It should be noted that only one OTU of type Id was found (OTU 15) among all the samples. Nevertheless, this OTU occurred in two habitats of the site C (Col_C_MM and Col_C_V_CT6) (**Figure 6**).

We attempted to identify the relationships between chemical parameters and MOB communities of the Congo sedimentary habitats using CCA and CoIA analysis. Both CCA and CoIA indicated no significant correlation (*p* > 0.05) with the selected chemical parameters (CH_4_ efflux, oxygen uptake, oxygen penetration depth) (Supplementary Table 1) and the MOB community.

## Discussion

### Comparison of Relative MOB Abundances between Recent and Abandoned Congo Lobe Complex

Relative MOB abundances assessed by FISH indicated the presence of intact type I and type II cells for all site C samples (recent lobe complex) and they accounted for up to 32% of the bacterial cells. The recent lobe complex is receiving turbiditic inputs and fresh terrestrial OM in the lobe sediments (Baudin et al., 2017b). Moreover, OM mineralization in the recent lobe sediments produces considerable CH_4_ efflux (Supplementary Table 1) (Khripounoff et al., 2015), comparable to cold seeps (Boetius and Wenzhofer, 2013), which could support MOB communities. In contrast, MOB cells were not detected in site E sediment (previous lobe complex), either with FISH or *pmoA* amplification attempts. Formerly connected to the present-day active channel, the previous lobe complex (site E) was disconnected after the avulsion of the active channel to the south that is currently feeding the recent lobe complex. It is believed that the previous lobe complex has not been affected by turbiditic sediment events for several 1000 years and is now fed with hemi-pelagic sedimentation mostly dominated by strongly oxidized marine OM with residual overflow of turbiditic inputs (Baudin et al., 2017b). The absence of recent turbiditic deposition might explain why CH_4_-depleted sediments are observed at site E (Supplementary Table 1) and thus, the absence of MOB cells in the previous lobe complex.

In all sedimentary habitats from the recent lobe complex, type I MOB cells were always around two- to three-fold more abundant than type II MOB cells, which is in agreement with previous studies reporting that type I cells are largely dominant in methane seeps (Niemann et al., 2006; Lösekann et al., 2007; Steinle et al., 2015). Moreover, the abundance of type I and type II MOB cells in Congo lobe sediments were two orders of magnitude higher than reported from the water column above methane seeps at the Svalbard continental margin (Steinle et al., 2015) and two orders of magnitude less abundant than those found in surface sediment at the center of HMMV, which is characterized as a very active seep poor in OM (Lösekann et al., 2007). This suggests that Congo deep-sea fan harbored relatively high abundance of MOB compared to cold seeps.

Among the habitats at site C (recent lobe complex), black patch sediments hosted the highest number of MOB cells (**Table 2**). MOB cells may be stimulated by high CH_4_ efflux (Supplementary Table 1) and by high TOC contents (>4.5%), characteristic of black patch habitats (Baudin et al., 2017b). Black patches were widespread in the recent lobe complex (Rabouille et al., 2016) and they likely correspond to areas where CH_4_ escapes from the sediment due to rapid diagenesis of OM. The black uniform color of the recovered sediments indicates an O_2_ depletion due to CH_4_ oxidation (Baudin et al., 2017b). In contrast, a lower abundance of type I cells and smaller aggregates formation in vesicomyid habitats (Col_C_V_CT6) might be due to high grazing pressure by feeding heterotrophic ampharetid polychaetes as previously shown in Hikurangi Margin seep sediments (Thurber et al., 2013).

### MOB Community Composition and Structure in the Congo Recent Lobe Complex

The representative sequences of MOB were not detected in the bacterial 16S rRNA gene libraries, indicating that the low sequencing effort (*n* = 100) using the cloning and Sanger sequencing based-method might not cover all the bacterial diversity. Previous studies were also unable to detect 16S rRNA gene sequences related to MOB with clone libraries but succeeded using high-throughput sequencing and *pmoA* functional gene clone libraries (Marlow et al., 2014).

Similarly, *pmoA* clone libraries showed that sedimentary habitats of the recent lobe complex hosted a great diversity of MOB lineages. However, general primer pairs used may not cover all the diversity of *pmoA* genes present in the Congo sediments. The design of revised general and new group specific primer pairs would improve the detection of *pmoA* genes, even amplify novel phylotypes. Most of the samples were dominated by type Ia sequences closely affiliated to the deep-sea 1 clade represented by the only cultivated member *Methyloprofundus sedimenti* WF1^T^. This obligate methanotroph was recently isolated from surface sediments (0–1 cm) in Monterey submarine canyon off the coast of California (1828 m below sea-level) close to a whale fall lying 500 m from a methane cold seep and 23 km from shore (Tavormina et al., 2015). This is in agreement with studies on sediments of hydrocarbon seeps on the continental margin of California (Tavormina et al., 2008), in the Gulf of Mexico (Yan et al., 2006) and off the coast of Japan (Inagaki et al., 2004) that have also revealed the dominance of diverse and novel type I methanotrophs.

Microbial mat habitat at site F was dominated by sequences related to the *Methylobacte*r-like clade. This habitat is characterized by the highest CH_4_ efflux (208.8 mmol m^-2^ d^-1^) measured and relatively high diffusive oxygen-uptake (9.9 mmol m^-2^ d^-1^) concomitant to low oxygen penetration (4 mm) (Supplementary Table 1). Several studies have shown that *Methylobacter* spp. are particularly active at low O_2_/high CH_4_ ratios at the oxic–anoxic interface of soils and freshwater sediments (Reim et al., 2012; Hernandez et al., 2015).

Interestingly, vesicomyid habitats harbored unique and the most diversified OTUs, and their sediment-water interfaces were colonized by diverse polychaetes (Rabouille et al., 2016). These bottom dwellers create burrows networks that provide conduits for the transport of O_2_ deeply into the anoxic zone of the sediment, stimulating the recycling of OM (Aller and Aller, 1998). Hence ventilated animal burrows and bioturbation by vesicomyid bivalves may help to prevent local O_2_ depletion and may provide diverse CH_4_ or O_2_ micro-niches for diverse MOB lineages. Furthermore, vesicomyid habitats exhibited most of the type IIa sequences observed, which were related exclusively to the *Methylocystis*-like clades. Type II MOB are prevalent in terrestrial environments (Knief, 2015) and often persist in inactive and robust resting stages under environmental stress (Whittenbury et al., 1970) that may provide a potential seed bank of high diversity (Ho et al., 2011b). Some *pmoA* sequences were closely related to *Methylogaea oryzae* E10^T^ (type Ib), a mesophilic methanotroph isolated from a rice paddy field growing on CH_4_ and methanol as sole carbon and energy sources (Geymonat et al., 2011). It has been shown that in a 2000 year-old paddy field, some species of type Ib and type II MOB emerged from the potential seed bank to become active when CH_4_ and O_2_ were available (Ho et al., 2011a). These findings suggest that some MOB might have been transported by alluvial sediment to the recent lobe complex where most turbidity currents die and lose the material held in suspension by turbulence (Khripounoff et al., 2003).

### Comparison of MOB Communities between Turbiditic and Seep Sediments

Molecular identification of *pmoA* sequences showed that turbiditic sediments of the recent Congo lobe complex harbored a greater diversity and richness of MOB than seep sediment from the center of HMMV (**Figures 4**, **6**). HMMV exhibited only two clades of type Ia closely related to the marine genera *Methylomonas methanica* S1^T^ (Whittenbury and Krieg, 1984) and *Methyloprofundus sedimenti* WF1^T^ (Tavormina et al., 2015) without type II members. Variations in diversity could be explained by the difference in the methane sources as previously reported. The central area of HMMV is connected to a deep gas reservoir and is thus impacted by high velocities of ascending methane-rich fluid flows, which episodically (1 or 2 per year) expels deep sediments enriched in CH_4_ and poor in OM (Niemann et al., 2006). As a result, the high upward flow increases CH_4_ concentrations in the uppermost oxic sediment horizon stimulating the growth of MOB and preventing the diffusion of sulfate into the sediment, hence inhibiting sulfate dependent anaerobic CH_4_ oxidation (Niemann et al., 2006). In contrast, the presence of deep reservoirs in the recent Congo lobe complex has not been demonstrated and the fluvial source is the main known contributor of the OM and CH_4_ efflux in the recent Congo lobe complex (Baudin et al., 2017b). This region is affected by strong episodic turbidity events (∼2 years, Heezen et al., 1964) with high terrestrial organic carbon content (3–5%) (Stetten et al., 2015) that creates disturbances and OM-rich areas (Rabouille et al., 2016; Baudin et al., 2017a). Localized intense remineralization of the OM during early diagenesis produces high CH_4_ efflux that may provide broad ecological niches and the potential for higher diversity of MOB communities. The high similarity between MOB lineages found at turbiditic sediments and cold seeps could support the concept of stepping stones (Smith et al., 1989) existing between diverse deep-sea ecosystems including deep-sea fans.

Turbiditic sediments from the recent Congo lobe complex harbored diverse MOB lineages within the type I-a, -b, and -d and type II-a subgroups that were affiliated mainly with *pmoA* sequences from marine environments (unclassified MOB sequences from methane seeps; deep-sea clades 1–3, 5) but also with *pmoA* sequences from aquatic (freshwater) and terrestrial (soils, rice paddies) environments. Elemental and isotopic analysis have shown that the nature of OM in the recent lobe complex is dominated (>80%) by a terrestrial source containing a mixture of higher plant debris and soil derived OM and that signatures of sediments from the recent lobe complex and from the Congo River were similar (Stetten et al., 2015; Baudin et al., 2017b). Furthermore, uncommon elevated values of a specific bacteriohopanepolyols (BHPs) produced by type I MOB were reported down to 115 mbsf in marine sediments located on the lower Congo fan (ODP Site 1075) (Talbot et al., 2014). It was further shown that the source of BHPs were tropical wetland systems and soils in the Congo river catchment (Spencer-Jones et al., 2015). The presence of these compounds have also been observed in high relative abundance in marine sediments of the Amazon shelf and deep-sea fan (Wagner et al., 2014). These observations support that turbiditic deposits of the Congo deep-sea fan host MOB communities of possible terrestrial origin.

## Conclusion

Both *pmoA* and relative cell abundances surveys indicate the presence of typical marine and terrestrial MOB phylogenetic lineages within the oxic–anoxic interface sediments of diverse organic-rich habitats in the recent Congo lobe complex. The predominant terrigenous origin of the OM pool and the detection of typical terrestrial MOB phylogenetic lineages at deep water depth (5000 m) is one of the most surprising results of this study. The comparison with collection of sediments from the Congo River and canyon as well as other fan systems would provide additional information regarding the origin of terrestrial guilds in the marine environment. Moreover, the assessment of active communities using microcosms enrichments, methane oxidation rates and metagenomic-isotope labeling experiments could widen our understanding concerning the origin and role of MOB in the functioning of the peculiar ecosystems of the Congo deep-sea fan.

## Author Contributions

SB designed experiments, acquired, analyzed and interpreted data, wrote manuscript. YM analyzed and interpreted data, revised manuscript. SG collected and analyzed sequence data. FL collected samples and sequence data, revised manuscript. AG designed experiments, collected samples and revised manuscript. LT designed experiments, analyzed data, revised manuscript and supervised SB project’s.

## Conflict of Interest Statement

The authors declare that the research was conducted in the absence of any commercial or financial relationships that could be construed as a potential conflict of interest. The reviewer SER and handling Editor declared their shared affiliation, and the handling Editor states that the process nevertheless met the standards of a fair and objective review.
