# Supplementary material for: Relative Abundance and Diversity of Bacterial Methanotrophs at the Oxic–Anoxic Interface of the Congo Deep-Sea Fan
Source: Front Microbiol. 2017 Apr 25;8:715. doi: 10.3389/fmicb.2017.00715 (PMC5403828; doi:10.3389/fmicb.2017.00715)
Supplement: Supplementary file 1 [file Table_1.pdf]

Supplementary Material

Relative abundance and diversity of bacterial methanotrophs at the oxic-anoxic interface of the Congo deep-sea fan.

Sandrine BESSETTE, Yann MOALIC, Sébastien GAUTEY, Françoise LESONGEUR, Anne GODFROY, Laurent TOFFIN*

***Correspondance:** Corresponding Author: Laurent.Toffin@ifremer.fr

1 Supplementary Data

1.1 Geochemical analyses

Pore-water analyses were performed on replicate sediment push-cores using Rhizon samplers and methane (CH_4) concentrations were measured as described in (Vigneron et al., 2013). CH_4 fluxes at sediment-water interface were determined *in situ* by using a *Calmar* benthic incubation chamber (Caprais et al., 2010) deployed on almost every investigated habitats by the ROV Victor 6000 as described in (Khripounoff et al., 2015). Diffusive oxygen (O_2) uptake and oxygen penetration depth (OPD) in the bottom water and in the sediment were measured *in situ* and analyzed as described in (Rabouille et al., 2009).

2 Supplementary Figures and Tables

2.1 Supplementary Figures

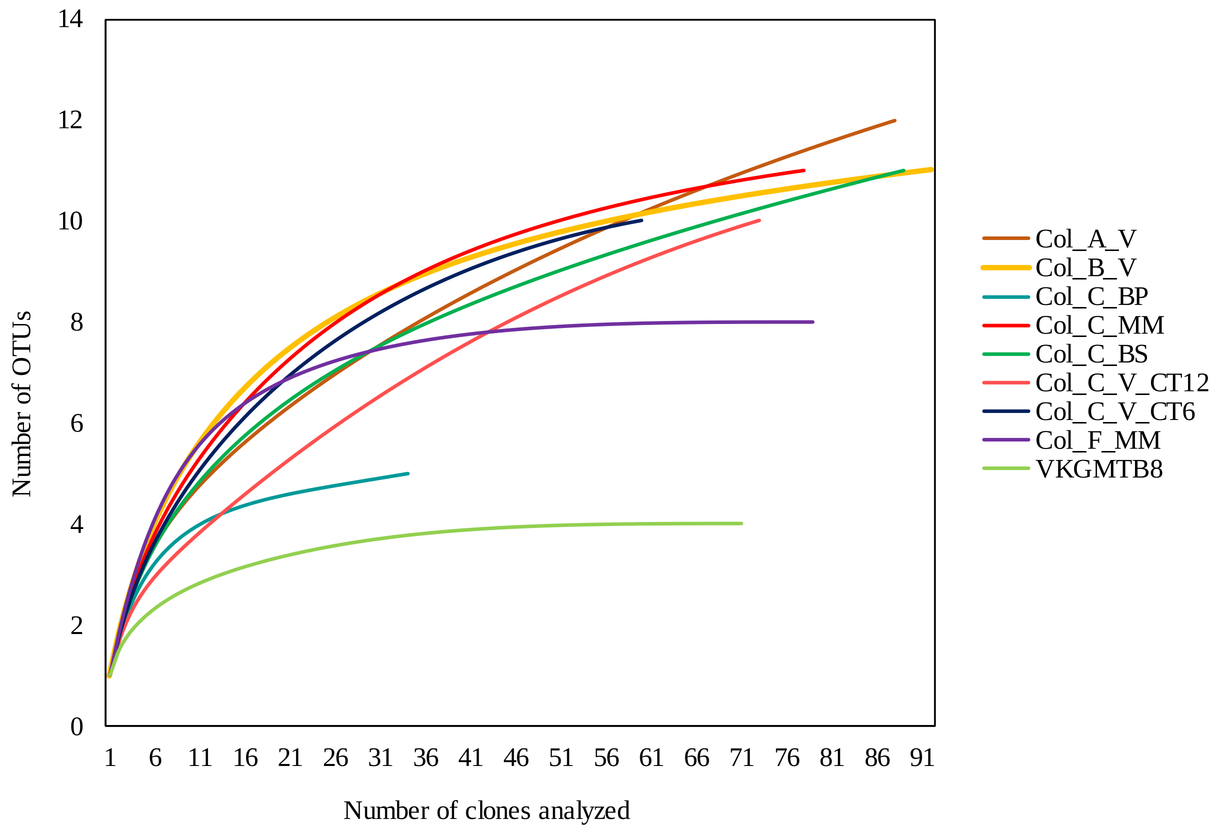

Supplementary Figure 1: Rarefaction curves for *pmoA* sequences clustered at 93% similarity in surface organic-rich and seep sediments of habitats in the Congo deep-sea fan (Col) and Haakon Mosby volcano (VKGMTB8) respectively. Samples are grouped color wise based on location.

Supplementary Table 1: Methane (CH_4) concentration, CH_4 efflux, diffusive oxygen uptake (DOU) and oxygen penetration depth (OPD) at sediment-water interface of different organic-rich sedimentary habitats of the Congo deep-sea fan. [(a)] data from (Pastor et al., 2017), [(b)] data from (Khripounoff et al., 2016), [(c)] data from this study and [(d)] (Pozzato et al., Submitted). Abbreviations: PL: Dive number; CT: Push-cores number, Col: **Co**ngo **l**obe. V: Vesicomyids habitats; BP: Black patch sediment; BS: Brown sediment; MM: Microbial mat habitats. NA: Not assessed during the Congolobe cruise.

Sites	Sample name	*Ex situ* CH_4 concentration (µM) [a]	*In situ* CH_4 efflux (mmol m^{-2} d^{-1})	*In situ* DOU (mmol m^{-2} d^{-1}) [d]	*Ex situ* OPD (mm) [d]
site A	Col_A_V	3.2	108.5 [b]	20.5	0.8
site F	Col_F_MM	143.6	208.8	9.9	3.9
site C	Col_C_MM	5.3	NA	11.5	1.56
	Col_C_BP	124.7	94 c	20.7	1.3
	Col_C_V_CT6	7.1	NA	19.4	1.6
	Col_C_V_CT12	NA	NA	8.3	2.2
	Col_C_BS	0.2	2.4 [c]	2.6	11
site B	Col_B_V	<0.1	8.4 [b]	2.8	9.4
site E	Col_E_BS	<0.1	0.0 [c]	1.4	66.5

Supplementary Table 2: List of FISH probes used in this study.

Name	Target group	Sequence (5'- 3')	reference
Eub338-Alexa	Most of Bacteria	GCT-GCC-TCC-CGT-AGG-AGT	(Amann et al., 1990)
MTMC701-Cy3	*Methylococcales*	GTG TTC CTT CAG ATC TCT	(Boetius et al., 2000)
Mγ84-Cy3	Type I MOB	CCA-CTC-GTC-AGC-GCC-CGA	(Eller et al., 2001)
Mγ705-Cy3	Type I MOB	CTG-GTG-TTC-CTT-CAG-ATC	(Eller et al., 2001)
Mα450-Cy3	Type II MOB	ATC-CAG-GTA-CCG-TCA-TTA-TC	(Eller et al., 2001)

3 References

Amann, R.I., Krumholz, L., and Stahl, D.A. (1990). Fluorescent-oligonucleotide probing of whole cells for determinative phylogenetic and environmental studies in microbiology. *J. Bacteriol.* 172**,** 762-770.

Boetius, A., Ravenschlag, K., Schubert, C.J., Rickert, D., Widdel, F., Gieseke, A., Amann, R., Jorgensen, B.B., Witte, U., and Pfannkuche, O. (2000). A marine microbial consortium apparently mediating anaerobic oxidation of methane. *Nature* 407**,** 623-626. doi: 10.1038/35036572.

Caprais, J.-C., Lanteri, N., Crassous, P., Noel, P., Bignon, L., Rousseaux, P., Pignet, P., and Khripounoff, A. (2010). A new CALMAR benthic chamber operating by submersible: First application in the cold-seep environment of Napoli mud volcano (Mediterranean Sea). *Limnology and Oceanography: Methods* 8**,** 304-312. doi: 10.4319/lom.2010.8.304.

Eller, G., Stubner, S., and Frenzel, P. (2001). Group-specific 16S rRNA targeted probes for the detection of type I and type II methanotrophs by fluorescence in situ hybridisation. *FEMS Microbiology Letters* 198**,** 91-97. doi: 10.1111/j.1574-6968.2001.tb10624.x.

Khripounoff, A., Caprais, J.C., Decker, C., Essirard, M., Le Bruchec, J., Noel, P., and Olu, K. (2015). Variability in gas and solute fluxes through deep-sea chemosynthetic ecosystems inhabited by vesicomyid bivalves in the Gulf of Guinea. *Deep Sea Research Part I: Oceanographic Research Papers* 95**,** 122-130. doi: 10.1016/j.dsr.2014.10.013.

Khripounoff, A., Caprais, J.C., Decker, C., Le Bruchec, J., Noel, P., and Husson, B. (2016). Respiration of bivalves from three different deep-sea areas: cold seeps, hydrothermal vents and organic carbon-rich sediments. *Deep Sea Research Part II: Topical Studies in Oceanography***,** in press. doi: 10.1016/j.dsr2.2016.05.023.

Pastor, L., Toffin, L., Decker, C., Olu, K., Cathalot, C., Lesongeur, F., Caprais, J.C., Bessette, S., Brandily, C., Taillefert, M., and Rabouille, C. (2017). Early diagenesis in the sediments of the Congo deep-sea fan dominated by massive terrigenous deposits: Part III – Sulfate- and methane- based microbial processes. *Deep Sea Research Part II: Topical Studies in Oceanography*. doi: 10.1016/j.dsr2.2017.03.011.

Pozzato, L., Cathalot, C., Berrached, C., Toussaint, F., Stetten, E., Caprais, J.C., Pastor, L., Olu, K., and Rabouille, C. (Submitted). Early diagenesis in the Congo deep-sea fan sediments

dominated by massive terrigenous deposits: Part I - Oxygen consumption and organic carbon mineralization using a micro-electrode approach. *Deep Sea Research Part II: Topical Studies in Oceanography*.
Rabouille, C., Caprais, J.C., Lansard, B., Crassous, P., Dedieu, K., Reyss, J.L., and Khripounoff, A. (2009). Organic matter budget in the Southeast Atlantic continental margin close to the Congo Canyon: In situ measurements of sediment oxygen consumption. *Deep Sea Research Part II: Topical Studies in Oceanography* 56**,** 2223-2238. doi: 10.1016/j.dsr2.2009.04.005.
Vigneron, A., Cruaud, P., Pignet, P., Caprais, J.-C., Cambon-Bonavita, M.-A., Godfroy, A., and Toffin, L. (2013). Archaeal and anaerobic methane oxidizer communities in the Sonora Margin cold seeps, Guaymas Basin (Gulf of California). *ISME J* 7**,** 1595-1608. doi: 10.1038/ismej.2013.18.
